# Fine structure of the cardiac muscle cells in the orb-web spider *Nephila clavata*

**DOI:** 10.1186/s42649-020-00030-x

**Published:** 2020-05-14

**Authors:** Yan Sun, Hyo-Jeong Kim, Myung-Jin Moon

**Affiliations:** Department of Biological Sciences, Dankook University, 119 Dandae-ro, Cheonan, 31116 South Korea

**Keywords:** Fine structure, Cardiac muscle, Sarcomere, Spider, *Nephila clavata*

## Abstract

The fine structural characteristics of cardiac muscle cells and its myofibril organization in the orb web spider *N. clavata* were examined by transmission electron microscopy. Although myofibril striations are not remarkable as those of skeletal muscles, muscle fibers contain multiple myofibrils, abundant mitochondria, extensive sarcoplasmic reticulum and transverse tubules (T-tubules). Myofibrils are divided into distinct sarcomeres defined by Z-lines with average length of 2.0 μm, but the distinction between the A-band and the I-bands is not clear due to uniform striations over the length of the sarcomeres. Dyadic junction which consisted of a single T-tubule paired with a terminal cisterna of the sarcoplasmic reticulum is found mainly at the A-I level of sarcomere. Each cell is arranged to form multiple connections with neighboring cells through the intercalated discs. These specialized junctions include three types of intercellular junctions: gap junctions, fascia adherens and desmosomes for heart function. Our transmission electron microscopy (TEM) observations clearly show that spider’s cardiac muscle contraction is controlled by neurogenic rather than myogenic mechanism since each cardiac muscle fiber is innervated by a branch of motor neuron through neuromuscular junctions.

## Introduction

Cardiac muscle is an involuntary, striated muscle that constitutes the main tissue of the heart. It is organized into the basic units of striated muscle fibrils known as sarcomeres (Craig and Padrón 2004). The cardiac muscle has attracted researchers to study essential properties that provide persistent rhythmic contraction throughout lifetime without experiencing muscle fatigue. Skeletal and cardiac muscle are similar in many ways, but there are important physiological differences that make the cardiac muscles less fatigued.

Previous studies showed evidences that the cardiac muscle was highly adapted to resistant to fatigue (Nelesen et al. 2008). In particular, it has a large number of mitochondria, enabling continuous aerobic respiration through oxidative phosphorylation and a good blood supply that provides nutrients and oxygen (Lyons et al. 2006). Since cardiac muscle tissues is responsible for transmission of action potentials and calcium during muscle contraction (Lehman and Szent-Györgyi 1975; Gordon et al. 2000), the intercalated discs connect multiple cardiac muscle cells in single syncytial unit to support rapid spread of action potentials and synchronized contraction of the myocardium (Gutstein et al. 2003).

Cardiac muscles appear to have light and dark bands due to the organization of the contractile sarcomere units of the myofibrils (Craig and Padrón 2004; Craig and Woodhead 2006). Similar to skeletal muscle, but cardiac muscle differs in several ways. While skeletal muscles are consisted of extremely long fibers resulting from progressive fusion of hundreds of cells (Sommer and Waugh 1978), cardiac muscles are a network of individual cells connected to each other via intercalated discs, enabling coordinated contraction of the myocardium (Goossens et al. 2007).

Arthropods have an open circulatory system which differs in both structure and function from the closed circulatory system found in humans and other vertebrates (Wirkner et al. 2013). Since the heart is a simple muscular tube that pumps blood into the body cavity with an open circulatory system (Foelix 2011), there a prediction that it will be less efficient than vertebrates. However, as one of the oldest groups of animals, arthropods also share a number of specific features that have evolved them to adapt to every habitat over the last 500 million years (Schmidt-Rhaesa et al. 1999). Therefore, some arthropods possess far superior traits and features to that of vertebrates.

Although characteristics of the cardiac muscles with open circulatory system are commonly reported in insects (Auber 1967; Hagopian and Spiro 1967) and crustaceans (Atwood 1967; Fahrenbach 1967), few reports examine the fine structural features of sarcomere organization (Johnson 1968; Franzini-Armstrong 1974; Sherman 1987; Choi and Moon 2003; Kim and Moon 2018). In this study, we describe the fine structural characteristic of the cardiac muscles of the golden orb-web spider *Nephila clavata*.

## Materials and methods

Specimens of the orb-web spider *Nephila clavata* L. Koch (Araneidae: Nephilidae) were collected at the campus of Dankook University, Cheonan, Chungnam, South Korea. All spiders were maintained under ambient conditions with natural lighting in wooden frames (40 × 40 × 10 cm) with glass plates front and back, and fed insects and water daily.

For histologic preparation, adult female spiders were anesthetized with CO_2_ and dissected under light microscope in a drop of spider Ringer’s solution consisting of 160 mM NaCl, 7.5 mM KCl, 4 mM CaCl_2_, 1 mM MgCl_2_, 4 mM NaHCO_3_, 20 mM glucose, pH 7.4 (Moon and Tillinghast 2013; Moon 2018).

The heart was fixed in 2.5% paraformaldehyde-glutaraldehyde fixative buffered with 0.1 M phosphate buffer solution (pH 7.4). Post-fixation was performed with 1% osmium tetroxide and washed several times with same buffer solution. The tissues were dehydrated with graded concentrations of ethanol and propylene oxide and embedded in Poly/Bed 812-Araldite mixture (Polysciences Inc., Warrington, PA, USA) for transmission electron microscopic examination.

Semi-thin sections, 0.5–1.0 μm thick, were obtained from a Leica Richert Ultracut R (Leica Microsystems, Wetzlar, Germany) using a glass knife. They were stained with 1% toluidine blue dissolved in 1% borax, and photographed using a Zeiss Axiophot microscope (Carl Zeiss, Jena, Germany) combined with a Motic digital imaging system (Motic Instruments Inc., Richmond, Canada).

Ultrathin sections were obtained using an Ultra 45° diamond knife (Diatome, Hartfield, PA, USA), and double stained with uranyl acetate and lead citrate solution. After these treatments, the sections were examined with a JEM 2100 Plus transmission electron microscope (JEOL, Tokyo, Japan) of the Korea Basic Science Institute (KBSI) at Ochang, Chungbuk, South Korea.

## Results

**Fig. 1:**
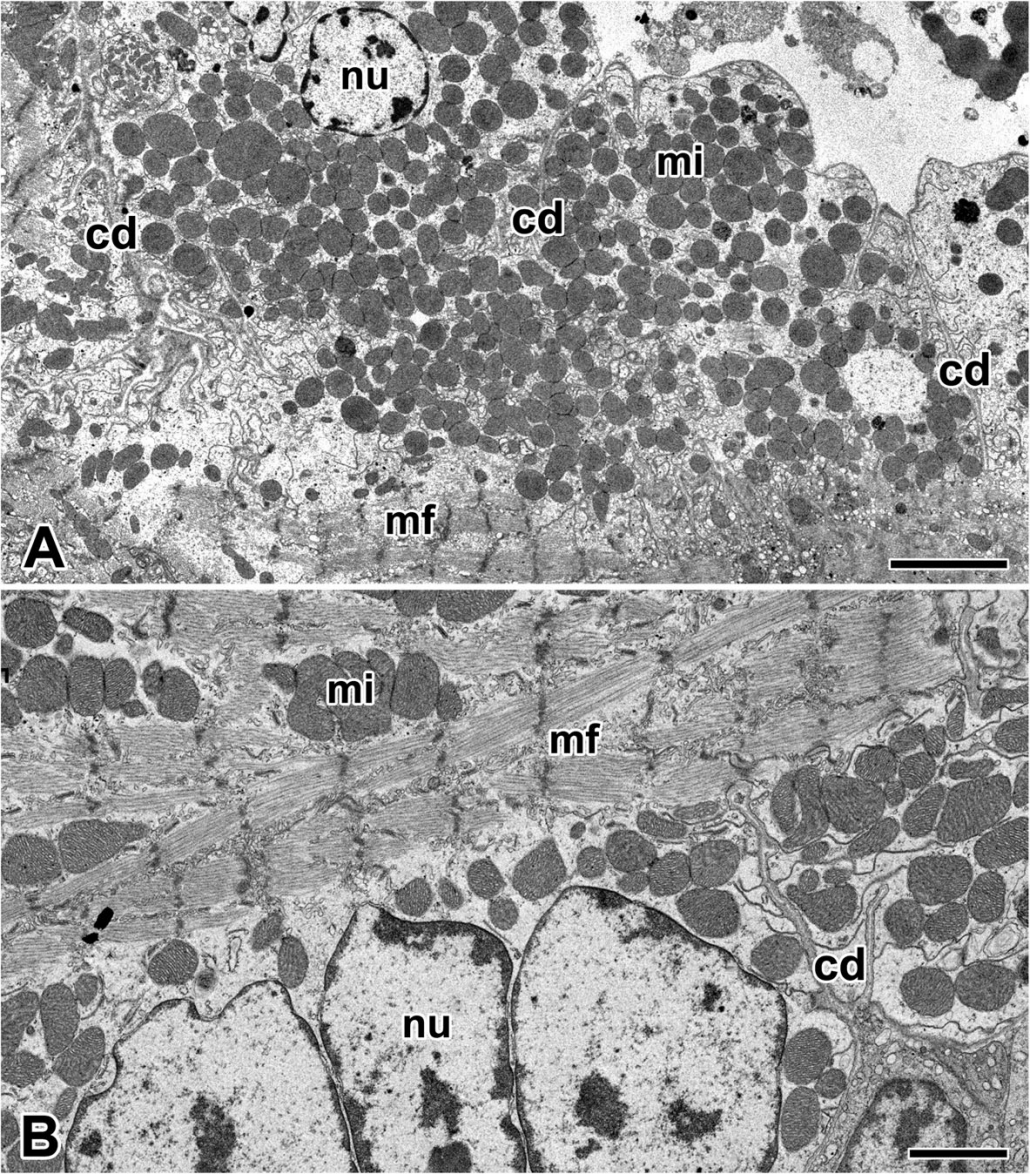
Electron micrographs of the cardiac muscle cells in the orb-web spider, *N. clavata*. **a**: Cardiac muscle cells contain densely packed mitochondria (mi) and myofibrils (mf) within the cytoplasm. Adjacent cells are tightly connected by intercalated disc (cd). **b**: Cardiac muscle cells show myofibril striations with distinct sarcomeres defined by Z-lines. Multinucleate cardiac muscle cell that has more than one nucleus (nu) per cell is seen. 5 μm (**a**), and 2 μm (**b**)

Spiders have an open circulatory system, and heart is basically a muscular tube with one-way valve on each end that pumps blood into the body cavity. In cross sections, cardiac muscles appear as finger-like projections into the heart lumen. The heart possesses no internal covering, instead the muscle is directly exposed to the hemolymph. Basically, cardiac muscle cells are striated and arranged in cylindrical form with densely packed myofibrils over the length. Each cell is arranged to form a multiple connection with neighboring cells via intercalated discs (Fig. 1a).

In *N. clavata*, morphology of the cardiac muscle is similar to that of skeletal muscles due to the arrangement of contractile myofilaments, but myofibril striations are not remarkable as those of skeletal muscles. In particular, there was only one nucleus per cell, but there were exceptions. A few multinucleate cardiac muscle cell that has more than one nucleus per cell is also seen (Fig. 1b).

**Fig. 2:**
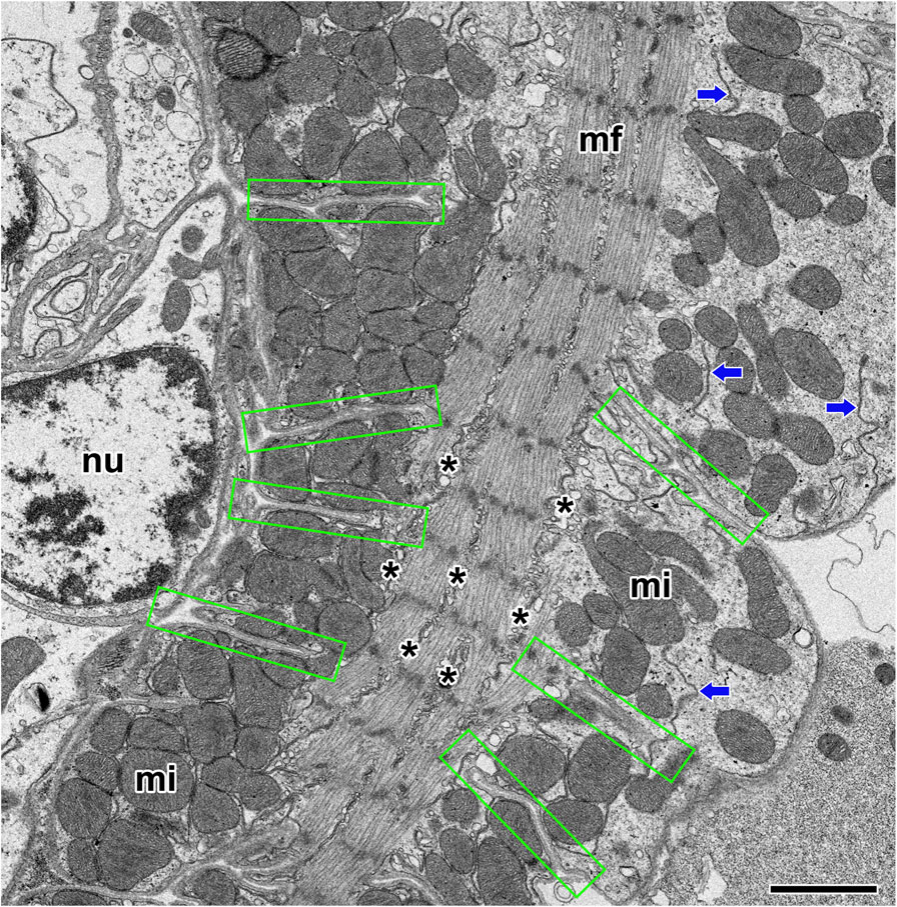
Electron micrograph of the cardiac muscle cells surrounded by scalloped edge of the sarcolemma (rectangles) in the orb-web spider, *N. clavata*. Each fiber contains multiple myofibrils (mf) and numerous mitochondria (mi), extensive sarcoplasmic reticulum (asterisks) and T-tubules (arrows). Note deeply penetrated plasma membrane (pm), and highly aggregated mitochondria. nu: nucleus. Scale bar indicates 2 μm

Cardiac muscle fiber has a highly organized internal structure for effective contraction. Each fiber contains multiple myofibrils and numerous mitochondria, extensive sarcoplasmic reticulum and T-tubules. The individual cardiac muscle cells are tightly connected to another cell by intercalated disc. The intercalated discs appear as thin, typically twig-like structures dividing adjacent cardiac muscle cells and running perpendicular to the direction of muscle fibers. In addition, each muscle cell is covered with a sarcolemma which consisted of a thin plasma membrane and a thick extracellular membrane. The extracellular membrane is directly adhered with the outer surface of the plasma membrane (Fig. 2).

**Fig. 3:**
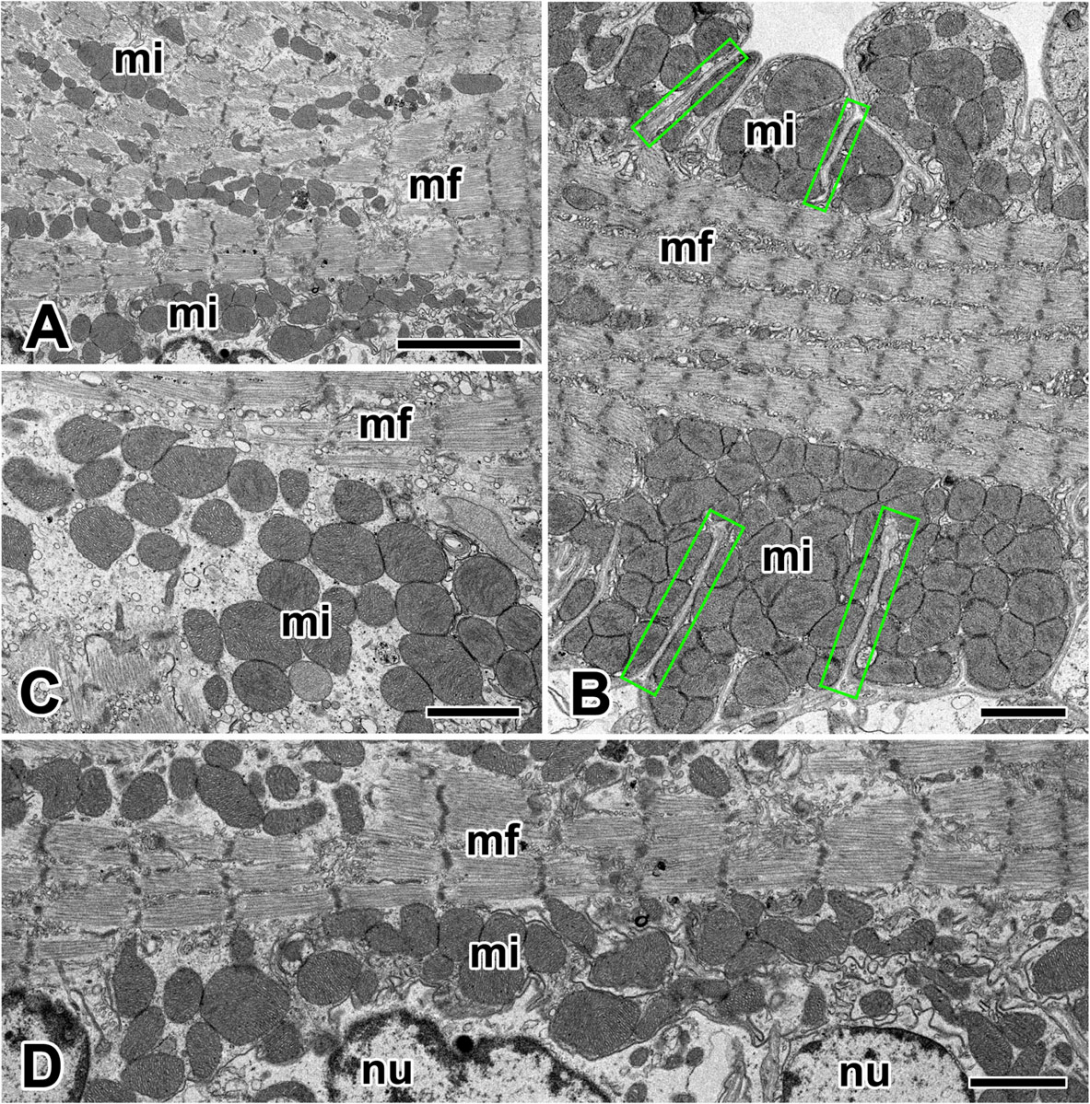
Electron micrographs of the cytoplasmic mitochondria in the cardiac muscle cells in *N. clavata*. **a**: Cardiac muscle cells have a lot of myofibrils (mf) and mitochondria (mi) in the cytoplasm. **b**: Mitochondria are located in the scalloped edge of the sarcolemma (rectangles) surrounding the myofibrils (mf). **c**: Oval or elliptical shaped mitochondria contains well developed cristae that provide an increase area of surface. **d**: Bundles of myofibrils and numerous mitochondria are distributed along the long axis of the muscle fiber. cd: intercalated disc, nu: nucleus. Scale bars indicate 5 μm (**a**) and 2 μm (**b-d**), respectively

In *N. clavata*, mitochondria with well-developed cristae were distributed around the nucleus of the cardiac muscle cells. Some of the sarcolemma deeply penetrated muscle fibers, and mitochondria are located in the clefts with highly aggregated pattern of arrangement (Fig. 3a). The mitochondria lying between myofibrils are particularly abundant, but a lot of mitochondrial aggregates are also found at junctional areas surrounded by scalloped edge of the sarcolemma (Fig. 3b).

Mitochondria of *N. clavata* cardiac muscle were found oval or elliptical in shape and range in variable sizes. The inner membrane of the mitochondrion is give rise to numerous cristae that provide an increase in the surface area (Fig. 3c). A muscle fiber is actually a cell whose cytoplasm contains bundles of myofibrils and numerous mitochondria. Mitochondria closely approach the intercalated discs, and they ran parallel to the long axis of the myofibrils (Fig. 3d).

**Fig. 4:**
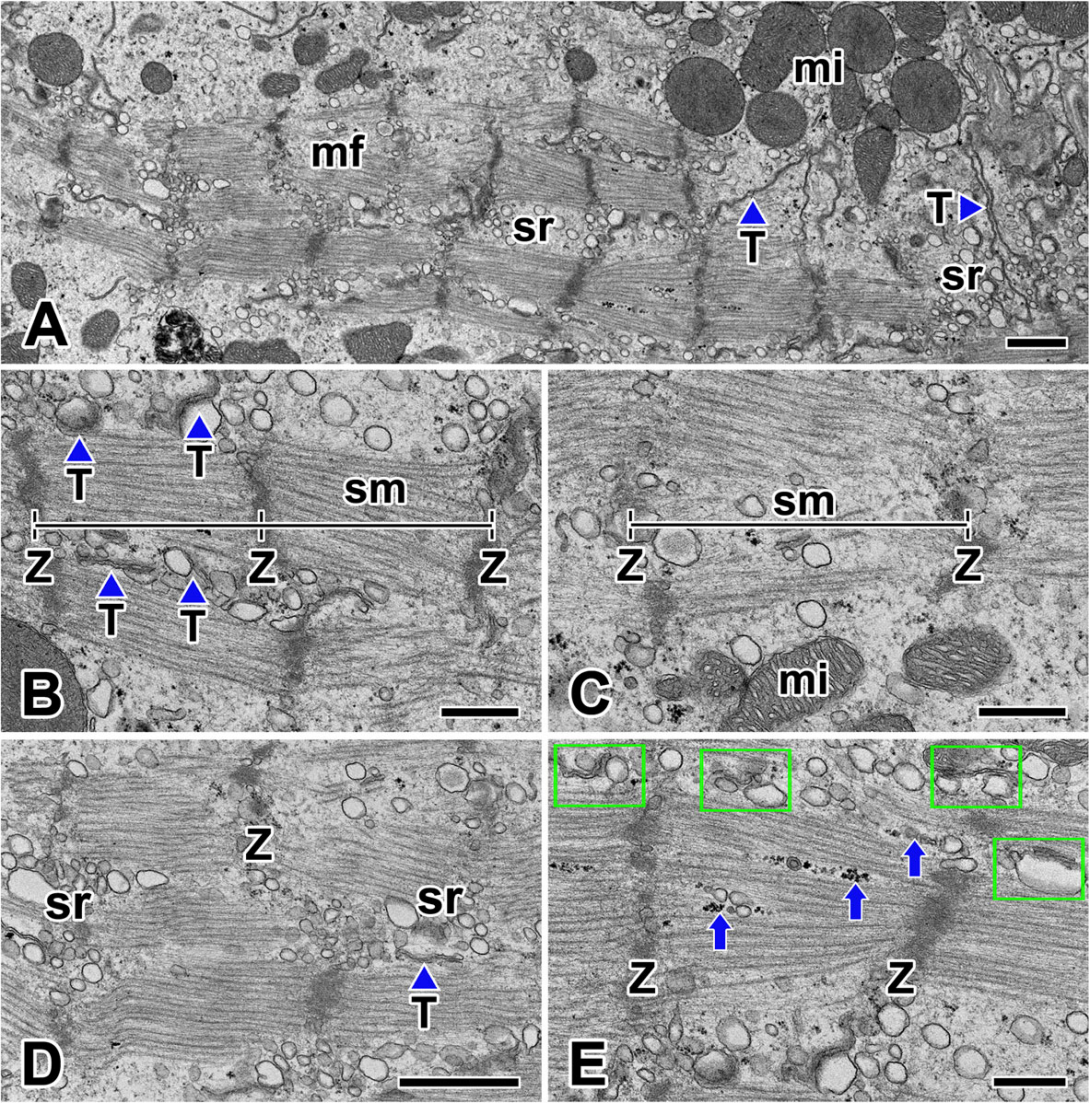
Electron micrographs of the myofibrils and sarcomere unit of cardiac muscle cells in *N. clavata*. **a**: Myofibrils (mf) show repeating units of sarcomere composed of thick and thin myofilaments. **b**, **c**: Each sarcomere (sm) is defined by adjacent Z-lines (Z). When muscle contraction occurs, A and I bands are not clear even at high magnification. **d**, **e**: Sarcoplasmic reticula (sr) are distributed around the myofibrils, and dyad (rectangles) structures which composed of a single t-tubule (T) and a terminal cisterna of the sarcoplasmic reticulum are seen near the Z-lines. Arrows indicate glycogen particles. mi: mitochondria. Scale bars indicate 1 μm (**a**, **b**) and 0.5 μm (**c-e**), respectively

In the cardiac muscle of *N. clavata*, myofibrils are divided into distinct sarcomeres defined by Z-lines. The sarcomere is the repeating units of a striated muscle which composed of thick and thin myofilaments. These filaments are overlapped in a regular repeating arrangement between a pair of Z-lines to form the sarcomere subunit. The Z-line is a dense fibrous structure, and the thin myofilaments are anchored at either side of the Z-line (Fig. 4a).

Longitudinal section of myofibril showed well defined contractile sarcomere with a length of 1.6 μm to 2.4 μm (average 2.0 μm). The discontinuous thick filaments are situated in the center of the sarcomere, but they do not extend to the Z-lines. Although the sarcomeres defined by the adjacent Z-lines is very apparent in shape, the distinction between the central A-band and the I-band adjacent to the Z-line is not clear even at high magnification. It was difficult to distinguish the H-zone because uniform striations of myofilaments were maintained over the length of sarcomeres (Fig. 4b, c).

Cardiac muscle fiber has numerous dyads or triads structures, visible in muscle fibers that have been sectioned longitudinally. Dyadic and a few triadic couplings are found mainly at the A-I level. The dyad structure is located near the sarcomere Z-line of the cardiac muscle cells. It is composed of a single T-tubule paired with a terminal cisterna of the sarcoplasmic reticulum (Fig. 4d). The T-tubules are formed by an invagination of the sarcolemma that extends into the center of the cell, and make contact with sarcoplasmic reticulum. The sarcoplasmic reticulum is a network of tubules that extend throughout muscle cells, wrapping around the myofibrils (Fig. 4e).

**Fig. 5:**
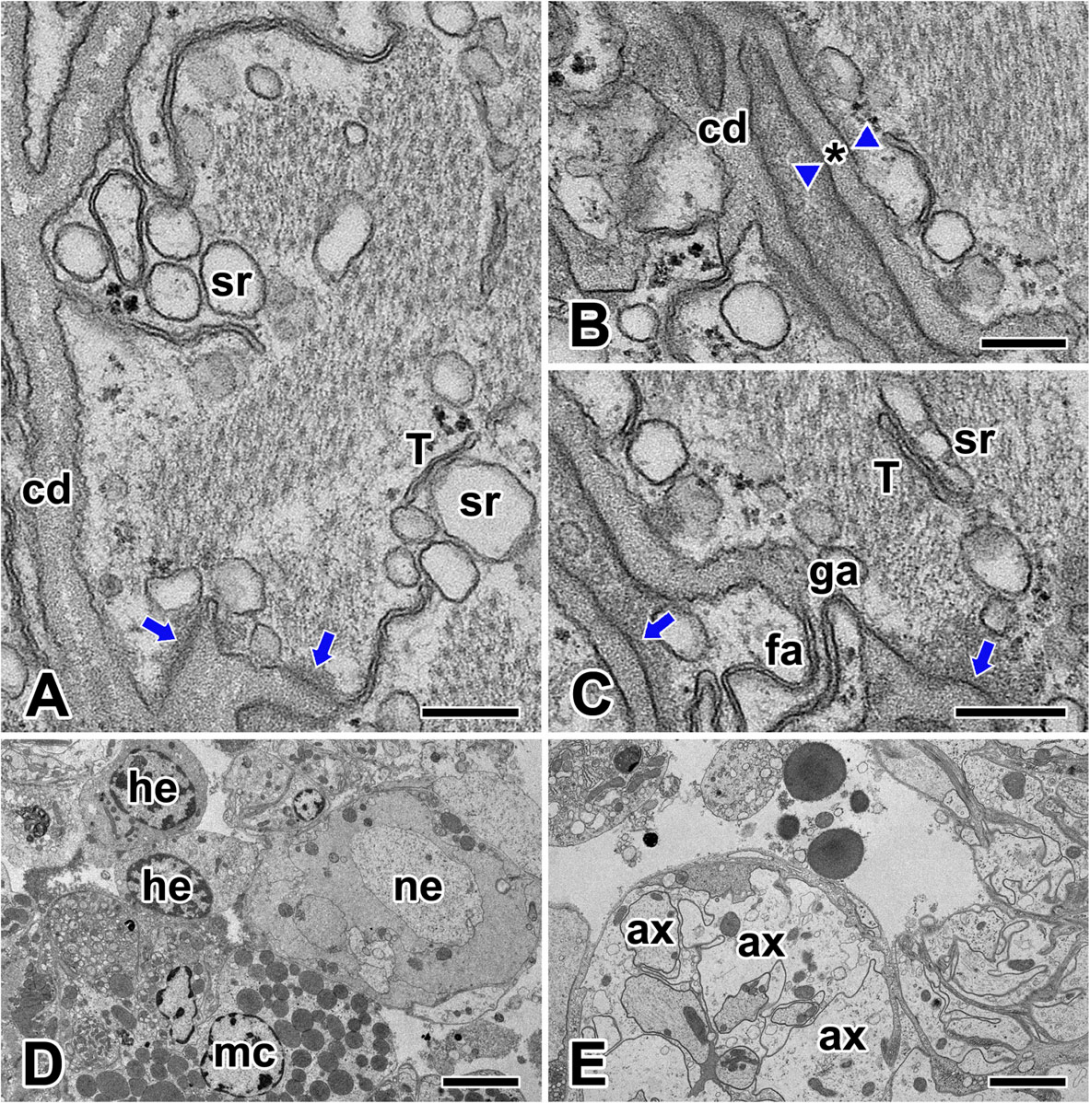
Electron micrographs of the intercalated disc and neural innervation of cardiac muscle cells in *N. clavata*. **a**: Intercalated disc (cd) is formed by deep penetration of the sarcolemma. Coupling of transverse tubules (T) and terminal cisternae of sarcoplasmic reticulum (sr) are observed. **b**: Intercalated discs are composed of dense layer of sarcolemma, two plasma membranes (arrowheads), and a regular interspace (asterisk). **c**: Gap junctions (ga), fascia adherens (fa), and macula adherens (arrows) junctions are seen. **d**, **e**. Cardiac muscle cell (mc) is innervated by a branch of external nerves (ne). A bundle of axons (ax) extend across the heart and penetrate the myocardium. Scale bars indicate 0.25 μm (**a-c**), 5 μm (**d**), and 2.5 μm (**e**), respectively

Under high magnification of electron microscopy, some of the sarcolemma deeply penetrated muscle fibers and formed numerous intercellular clefts. It was also observed that intercalated discs are extending between the myofibrils longitudinally and transecting the myofibrils transversely (Fig. 5a). They are composed of several layer which are the dense inner layer of the sarcolemma, two plasma membranes, and a regular interspace (Fig. 5b). The intercalated disc of the cardiac muscle in the spider *N. clavata* contains abundant gap junctions which have a continuous narrow junctional membrane. Moreover, another two types of intercellular junctions, fascia adherens and macula adherens (desmosomes), were also found (Fig. 5c).

Each cardiac muscle fiber is innervated by a single branch from a motor neuron, and this branch forms a neuromuscular junction with the muscle cell membrane (sarcolemma). In *N. clavata*, axon branches through the muscle and connects to the individual muscle fibers at the neuromuscular junction. Impulses arriving on the nerve fiber are transmitted to the sarcolemma and ultimately cause the contraction of the muscle fiber (Fig. 5d, e).

## Discussion

In vertebrate animals, three distinct layers of tissue make up the heart wall: the epicardium, myocardium, and endocardium (Gordon et al. 2000). Among them, the endocardium is a delicate layer of membranous tissue that lines the heart and blood vessels. However, the heart possesses no endocardial tissues in the spider *N. clavata*, instead the muscle is directly exposed to hemolymph. This is a clear difference between the fine structural properties of the spider cardiac muscle and that of the vertebrate (Fawcett and McNutt 1969; Hoyle 1969). The reason is that the spider has an open circulatory system, so there is no capillary network surrounding the muscle fibers in the connective tissue layer of the heart (Foelix 2011).

The myocardium is thick contractile middle layer constructed cardiac muscle cells. In vertebrates, tunicates, and some molluscs, the heart beat is initiated and regulated by specialized groups of muscle cells, myocardial conducting cells. Although they are one of specialized cardiac muscle cells, the myocardial conduction cells initiate the action potential myogenically (Gordon et al. 2000; van Weerd and Christoffels 2016). But it has been reported that some invertebrates including insects, heart contraction is initiated and regulated by external nerve (Sherman 1987). Our TEM observation clearly shows that the cardiac muscle fiber of this spider is innervated by a branch of external nerves through neuromuscular junctions. It means that this spider’s heart is not myogenically driven but neurogenically driven.

Previous studies have shown that the vertebrate cardiac muscle tissue is composed of many branching cells that are joined into a continuous mass by intercalated discs (Franke et al. 2006; Goossens et al. 2007). In *N. clavata,* cardiac muscle cells are also striated and each cell is arranged to form a multiple connection with neighboring cells. The intercalated disc is situated at the end of cardiac muscle cells and provides the electrochemical and mechanical connection between neighboring cells (Bennett 2018). It also possesses functional significance that enable the rapid transmission of electrical impulses, enabling the myocardium to act in a coordinated contraction (Forbes and Sperelakis 1985).

Our TEM observation also demonstrates that the intercalated discs of spider cardiac muscle are composed of several layer and a regular interspace. In particular, intercalated disc contains abundant gap junctions and two types of intercellular junctions. This is consistent with previous reports that the intercalated discs are composed of three different types of cell to cell contacts: adherens junctions, desmosomes and gap junctions (Franke et al. 2006; Ehler 2016). It has been demonstrated that individual cardiac muscle cells are connected by intercalated discs to work as a single functional syncytium. The advantage of the syncytium structure is that the cardiac fibers form a continuous sheet of muscle and the muscle cells can pass an action potential along a large area of heart wall (Ehler 2016). Thus the encircling myocardium can compress the heart cavities with great force.

In *N. clavata*, cardiac muscle cells have numerous mitochondria to fulfil the high energy requirements of the cardiac contraction. Recently, Kim and Moon (2018) also showed abundant mitochondria occupying more than 30% of cytoplasmic volume of the cardiac muscle cells of black widow spider *Latrodectus mactans*. This is consistent with our current prediction that mitochondrial content in muscle fibers reflects the need for continuous aerobic metabolism during vigorous contraction of cardiac muscles (Dirksen 2009).

In addition, mitochondria detected by electron microscopy, were clustered around the nucleus, between the myofibrils and beneath the sarcolemma. Previous study has shown that mitochondrial abundance can greatly differ depending on fiber types, physiological and environmental conditions (Leary et al. 2003), and aging related changes (Hood 2001; Lyons et al. 2006). In particular, mitochondria clustered around the nucleus indicate the high-energy requirements of nuclear processes and the necessity to keep the distance of energy transfer from mitochondria to the nucleus as short as allowed by cellular architecture (Dzeja et al. 2002).

Sarcomere is the basic structural and functional unit of the myofibril in both skeletal and cardiac muscle (Craig and Padrón 2004; Zoghbi et al. 2008). In *N. clavata*, each fiber contains tightly packed myofibrils which run the length of the fiber and are composed of repeating sections of sarcomeres. Thick filaments are anchored in the middle of the sarcomere, and the thin filaments are the region on either side of a Z-line. This partial overlap in filaments makes alternating dark and bright areas known as the A-band and I-band (Fawcett and McNutt 1969; Zoghbi et al. 2008).

Although defined length of contractile sarcomere in *N. clavata* is measured of 1.6 μm (contracted muscle) to 2.4 μm (relaxed muscle) at longitudinal section of myofibril, distinction between the A-band and I-band is not clear even at high magnification. It is also difficult to distinguish the H-zones and M-lines because of uniform striations of whole sarcomeres. Previous studies have shown that cardiac muscles of horseshoe crab (Leyton and Sonnenblick 1971) and black widow spider (Kim and Moon 2018) were characterized by long sarcomeres and greater length of thick filaments. Both traits are expected to provide an increased number of cross-bridges in the sarcomeres.

Previous studies have shown that the sarcomere length are associated with several factors, such as the architecture of the Z-line, the length of A-band and I-bands, diameter of thick filament, and ratio of thin and thick filaments (Leyton and Sonnenblick 1971; Gordon et al. 2000). This is consistent with our observation in the cardiac muscles of *N. clavata*. Our electron microscopic examination of the sarcomere length of contracted muscle revealed approximately 2 μm representing characteristics of fast muscle. This is particularly true because it has been reported that the sarcomere length of fast muscles, such as insect flight muscles, is 2 to 4 μm long, whereas slow muscles of legs and body walls tend to expand from 7 to 10 μm (Leyton and Sonnenblick 1971; Cutts 1988).

T tubules or transverse tubules are tube-like invaginations of a plasma membrane (sarcolemma). Sarcomeres are connected to the sarcolemma by T-tubules which speed up the rate of depolarization within the sarcomere (Ehler 2016). T-tubules are closely associated with a specific region of the sarcoplasmic reticulum (SR), known as the terminal cisternae in skeletal and cardiac muscle (Franke et al. 2006). It has been known that this is the primary site of calcium release (Franzini-Armstrong 1974). In *N. clavata*, the dyad structure of the cardiac muscle cell is located near the either side of sarcomere Z-lines. It is composed of a single T-tubule paired with a terminal cisterna of the sarcoplasmic reticulum. They occur at regular intervals along the muscle fiber and extend inward with two dyads per sarcomere.

The dyad or triad structure plays an important role in excitation-contraction coupling by juxtaposing an inlet for the action potential near a source of calcium ions. It has been reported that the number of triads per sarcomere varies depend on species such as one per triad in frog and two triads in mammals. But, in fishes and crustaceans, only one cisterna of SR is associated with each T-tubule, thus forming a dyad instead of triad (Hagopian and Spiro 1967). The results of this study, together with comparisons to previous studies of the spider cardiac muscles, contribute additional knowledge of these specific arrangement of sarcomere and their cellular organelles for muscle contraction.

## Conclusion

This study was designed to understand the fine structural characteristics of spider cardiac muscle cells controlled by an open circulatory system. The cells are connected by the intercalated disc to work as a functional syncytium, and three types of intercellular junctions for coordinated contraction are also observed. We could observe myofibril organization with repeating units of sarcomeres which composed of two types of myofilaments. Striated appearance of the sarcomeres, including sarcomere length, Z-line, A and I bands showed similarities to other arthropods. However, the dyadic junctions of the t-tubule paired with the terminal cisterna of the sarcoplasmic reticulum showed substantial properties since two dyads were found per each sarcomere. Moreover, this study clearly shows that cardiac contraction is regulated by external nerves rather than myogenic control.

## Data Availability

Materials described in the manuscript, including all relevant raw data, will be freely available to any scientist wishing to use them for non-commercial purposes.

## Abbreviations

A-band: Anisotropic band
I-band: Isotropic band
SR: Sarcoplasmic reticulum
TEM: Transmission electron microscopy
T-tubule: Transverse tubule
